# Decreased survival among lung cancer patients with co-morbid tuberculosis and diabetes

**DOI:** 10.1186/1471-2407-12-174

**Published:** 2012-05-11

**Authors:** Shwn-Huey Shieh, Janice C Probst, Fung-Chang Sung, Wen-Chen Tsai, Ya-Shin Li, Chih-Yi Chen

**Affiliations:** 1 Department of Health Services Administration, China Medical University, Taichung, 40402, Taiwan; 2 Department of Nursing, China Medical University Hospital, Taichung, 40402, Taiwan; 3 Department of Health Services Policy and Management, Arnold School of Public Health, University of South Carolina, Columbia, SC, USA; 4 Department of Public Health, China Medical University, Taichung, 40402, Taiwan; 5 Department of Medical Research, China Medical University Hospital, Taichung, 40402, Taiwan; 6 Department of Health Systems Management, Chung-Shan Medical University and Hospital, Taichung, 40201, Taiwan; 7 Department of Respiratory Therapy, China Medical University, Taichung, 40402, Taiwan; 8 Cancer center, China Medical University Hospital, 2 Yu Der Road, Taichung, 40402, Taiwan

**Keywords:** Lung cancer, Comorbidity, Diabetes, Survival, Tuberculosis

## Abstract

**Background:**

Comorbid conditions influence the survival of cancer patients. This study evaluated the influence of comorbidity on survival among lung cancer patients.

**Methods:**

The authors evaluated the medical records of 1111 lung cancer patients of a medical center in Taiwan. Days of survival were calculated for each patient and mortality hazard ratios were estimated for associations with demographic status, comorbidity and cancer stage at diagnosis.

**Results:**

On average, the survival time was slightly longer among women than among men (838 ± 689 vs. 749 ± 654 days, *p* = 0.050). Survival days increased with age (from 580 ± 526 [≤ 50 years] to 803 ± 693 [≥ 71 years] days, *p* = 0.020) and decreased with stage (from 1224 ± 656 [stage I] to 489 ± 536 [stage IV] days, *p* < 0.001). Younger patients were more likely to be diagnosed with lung cancer at a late stage. Compared with lung cancer patients without tuberculosis, those with tuberculosis had a significantly shorter average survival duration (584 vs. 791 days, *p* = 0.002) and a higher mortality hazard ratio (1.30, 95% CI: 1.03 - 1.65). A similar trend was observed in lung cancer patients with diabetes.

**Conclusions:**

Lung cancer patients with comorbid tuberculosis or diabetes are at an elevated risk of mortality. These patients deserve greater attention while undergoing cancer treatment.

## Background

Cancer is a highly complicated disease. Cancer survival mainly depends on patient characteristics, the histology and pathology of the tumor, stage at diagnosis, host-tumor interaction, and comorbidities. Comorbidity has an inherent influence on each patient’s initial treatment and the treatment effectiveness of patient care. Previous studies have demonstrated that less aggressive treatment is given to patients with breast cancer, prostate cancer, lymphoma, or lung cancer who have specific existing comorbidities [1-6]. Several diseases such as hypertension, ischemic heart disease, cerebrovascular disease, chronic obstructive pulmonary disease (COPD), and diabetes mellitus (DM) are considered to have a significant influence on the survival of cancer patients [2,7-10].

In the case of lung cancer patients, pulmonary and cardiovascular function may have a significant impact on survival [2,11-14]. Elderly patients with Stage I or II lung cancer are less likely to receive surgery than younger patients [13]. Patients with COPD, cardiovascular disease, or DM comorbidity also have a lower resection rate [13]. Janssen-Heijnen et al. reported that the morbidity and mortality of non-small cell lung cancer (NSCLC) patients following resection are associated with poor pulmonary function or cardiovascular disease [2]. Older NSCLC patients have a higher prevalence of comorbid cardiovascular disease or COPD, which may cause additional morbidity and reduce their survival. Battafarano et al. indicated that NSCLC patients with comorbidity have a two-fold increased risk of death compared with patients without comorbidity [11].

The presence of multiple comorbid diseases is common among lung cancer patients, with 22.1% of patients having five or more comorbid diseases, 54.3% having three or more, and 88.3% having one or more [14-16]. Tammemagi et al. have reported that tuberculosis (TB), COPD, and DM are the most common comorbidities associated with a reduced survival among patients with lung cancer [14]. They also identified that comorbidity is important for predicting the survival of both localized and advanced lung cancer [16].

The symptoms of lung cancer can be masked by the symptoms of comorbid diseases such as chronic bronchitis, COPD, TB, DM, hypertension (HT), or even heart disease [15,17,18]. Patients with comorbid diseases may ignore symptoms or delay reporting them to a physician, because the symptoms of lung cancer are often confused with those of comorbid diseases. Comorbid diseases may exert direct effects on the host immune system and reduce the duration of survival, and are thus among the most important factors for determining lung cancer survival [19,20].

The objective of this study was to investigate the influence of comorbidity on the survival of patients with lung cancer. Furthermore, we presented data showing the associations between selected comorbid diseases (TB, DM, HT, COPD, and other cancer [OC]) and survival.

## Materials and methods

### Sample

Data on the care of 1410 patients newly diagnosed with histologically confirmed lung cancer between October 1997 and December 2004 at a medical center in central Taiwan were extracted from the medical records of 2516 lung cancer patients by three trained medical nurses. Group I included all lung cancer patients with resectable tumors (n = 626) who had received surgery at the Department of Thoracic Surgery. Group II comprised 784 patients with late-stage lung cancer who were randomly selected from 1890 patients cared for at the Departments of Chest Medicine and Radiotherapy. There were no significant differences between the 784 randomly selected patients and those not selected in terms of age (*p* = 0.309) or sex (*p* = 0.804).

Among the 1410 patients in groups I and II, 299 were excluded from the analysis because they had incomplete baseline information (for example, missing personal ID or unknown cancer stage), metastatic cancer from other organs or postoperative deaths. The remaining 1111 patients were included in the data analysis. This study was approved by the Institutional Review Boards of the medical center.

### Variable definitions

#### Survival

The number of days lived after the initial diagnosis was recorded. Patients’ vital status information was obtained from the official death registry. Patients were followed for more than 7 years or until deceased.

#### Comorbidity

Comorbidity was the disease present at the time of lung cancer diagnosis. We adopted the method developed by Charlson et al. to select comorbidities with potential association with lung cancer survival. These were OC, TB, DM, HT, and COPD [21].

#### Stage of the disease

The stage at diagnosis of each lung cancer case was defined in accordance with the classification outlined in the American Joint Committee on Cancer’s Cancer Staging Manual [22]. In Stage I, the cancer is in the lung only, with normal tissue around the tumor. In Stage II, the cancer has spread to nearby lymph nodes or the chest wall, diaphragm, mediastinal pleura, or parietal pericardium. In Stage III, the cancer has either spread to the lymph nodes in the mediastinum (N2; Stage IIIa) or to the lymph nodes on the opposite side of the chest or in the lower neck (N3; Stage IIIb). Stage III is locally advanced lung cancer. For the purpose of this study, patients with Stage IIIa or IIIb lung cancer are combined into a single group. In Stage IV, the cancer has spread to other parts of the body or to another lobe of the lungs. A physician checked the pathology or cytology reports and the clinical image studies to confirm the tumor stage diagnosis.

#### Control variables

Patients’ demographic characteristics (age, sex, religion, education, marital status, and occupation) with implications for survival were controlled for in the multivariate analyses.

### Data analysis

Data analyses first used descriptive statistical analyses to identify the mean, median and interquartile ranges of survival duration by sociodemographic status, comorbidity and cancer stage at diagnosis. Survival duration was compared between men and women, among age groups (≤ 50, 51–60, 61–70 and ≥ 71 years), other demographic variables, among patients with and without the selected comorbidity (OC, TB, COPD, DM and HT), and among lung cancer stages. Mean days of survival were also estimated to evaluate the interactions between comorbidities and cancer stages. Multivariate Cox proportional hazards regression was used to compute the adjusted lung cancer mortality hazard ratios (HR) and 95% confidence intervals (CI). Lung cancer mortality HRs were calculated separately for men and women, different age groups, patients with or without a specific comorbidity and different cancer stages. We also used a Kaplan–Meier model to compare patient survival rates between those with and without a comorbidity that was significantly associated with the duration of survival. Analyses were performed using the SAS Statistics System (Version 9.1, SAS Institute Inc., Cary, NC).

## Results

### Comparison of survival duration

The patients included in the analysis were mostly male, elderly, and married. Most patients had a low educational attainment and a high proportion of religious affiliation (Table 1). Overall, the mean survival duration was 772 ± 665 days, with a median of 624 days and interquartile range of 205–1190 days. Survival medians were consistently lower than survival means for all investigated variables. Survival duration was longer in women than in men, and increased with age, with mean from 580 ± 526 days for ≤ 50 years group to 803 ± 693 days for ≥ 71 years group (*p* = 0.020). Survival duration was not significantly associated with education level, religion or job status. Table 1 also shows that almost half the patients had at least one comorbid disease at baseline. The most prevalent comorbidity was HT (26.2%), followed by DM (11.7%), TB (9.7%), OC (5.8%) and COPD (5.0%). On average, patients with comorbid OC or COPD had a longer duration of survival, but this was not a statistically significant difference. Those with comorbid tuberculosis (p = 0.002), diabetes (p = 0.001) or hypertension (p = 0.178) had a shorter duration of survival. Patient survival duration decreased with advancing cancer stage, from an average of 1224 days for those diagnosed with stage I to 489 days for those diagnosed with stage IV cancer.

**Table 1:** Means, medians and interquartile ranges of survival days in patients with lung cancer by patient characteristics, co-morbidity and stage of disease

**Criteria**	**N = 1111**		**Survival Days**			***P*-value for means**
	**n**	**%**	**Median**	**25th - 75th**	**Mean ± SD**	
**Overall**	1111	(100)	624	205 - 1190	772 ± 665	
**Age**						
≤ 50	94	(8.5)	419	188 - 864	580 ± 526	0.020
51 - 60	155	(14.0)	597	234 - 1039	738 ± 641	
61 - 70	290	(26.1)	699	244 - 1228	792 ± 651	
≥ 71	572	(51.5)	654	187 - 1268	803 ± 693	
**Gender**						
Male	822	(74.0)	596	188 - 1152	749 ± 654	0.050
Female	289	(26.0)	711	259 - 1279	838 ± 689	
**Marital Status**						
Married	964	(88.1)	626	216 - 1200	780 ± 666	0.112
Single (included divorce)	130	(11.9)	529	123 - 971	682 ± 636	
**Education**						
< Junior high	654	(63.3)	556	185 - 1187	756 ± 681	0.397
Junior high	137	(13.3)	647	211 - 1163	751 ± 611	
Senior high	171	(16.6)	697	271 - 1287	842 ± 681	
≥ College	71	(6.9)	528	201 - 1115	707 ± 623	
**Religion**						
No	294	(28.0)	651	182 - 1163	763 ± 662	0.847
Yes	755	(72.0)	615	211 - 1193	772 ± 666	
**Occupation**						
Work	323	(30.0)	554	188 - 1122	744 ± 662	0.378
Retired	296	(27.5)	555	177 - 1170	743 ± 672	
Not work	457	(42.5)	715	233 - 1202	801 ± 654	
**Comorbidity**						
Other cancer						
No	1031	(94.2)	606	201 - 1180	764 ± 666	0.116
Yes	64	(5.8)	813	350 - 1376	898 ± 643	
Tuberculosis						
No	989	(90.3)	653	220 - 1206	791 ± 667	0.002
Yes	106	(9.7)	364	107 - 856	584 ± 612	
COPD						
No	1040	(95.0)	614	206 - 1176	765 ± 662	0.162
Yes	55	(5.0)	863	317 - 1353	894 ± 714	
Diabetes						
No	967	(88.3)	651	220 - 1220	794 ± 670	0.001
Yes	128	(11.7)	427	134 - 929	600 ± 601	
Hypertension						
No	808	(73.8)	654	220 - 1219	788 ± 660	0.178
Yes	287	(26.2)	>546	169 - 1122	726 ± 677	
**Stage**						
I	216	(19.4)	1164	743 - 1681	1224 ± 656	<0.001
II	106	(9.5)	913	546 - 1498	1045 ± 674	
III	406	(36.5)	555	220 - 1073	727 ± 622	
IV	383	(34.5)	273	94 - 723	489 ± 536	

COPD: chronic obstructive pulmonary disease.

### Survival duration by comorbidity and cancer stage

**Figure 1:**
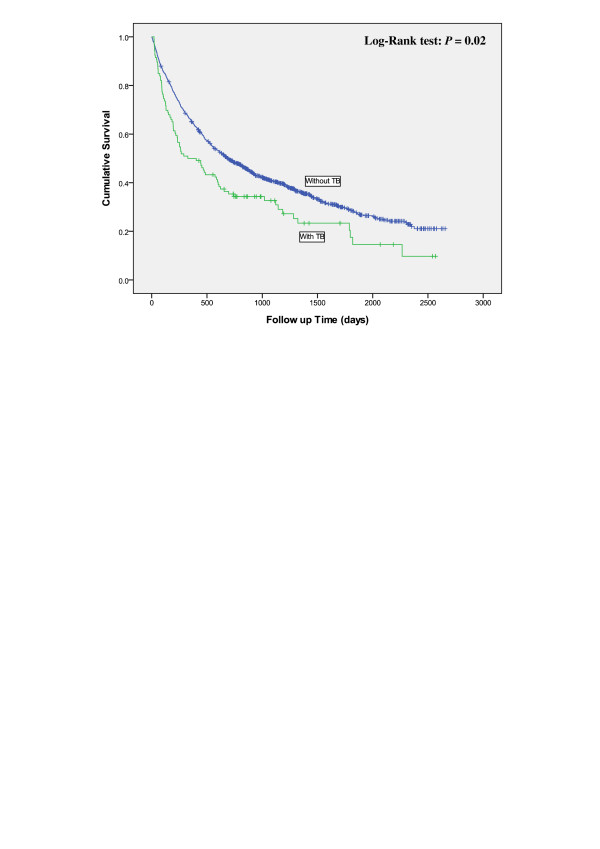
The comparison of survival time between lung cancer patient with or without TB.

**Figure 2:**
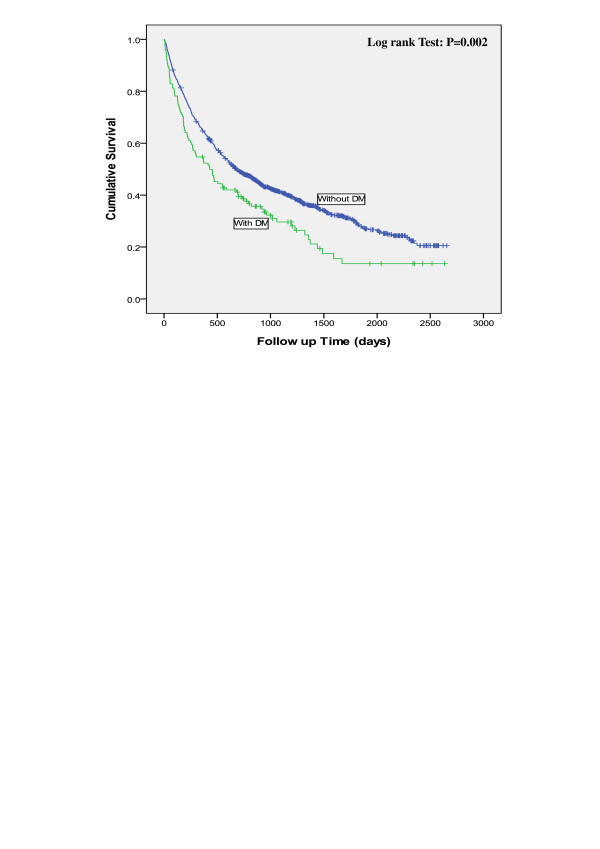
The comparison of survival time between lung cancer patient with or without DM.

Table 2 shows the mean survival duration for lung cancer patients by comorbidity and cancer stage. In general, the mean survival duration decreased with advancing cancer stage. Patients with a comorbidity tended to have a shorter survival duration, except for those with comorbid COPD or HT who were diagnosed with lung cancer at an early stage. On average, patients with comorbid TB or DM had a shorter survival duration than patients without the corresponding diseases among all stages. The Kaplan–Meier analysis showed that 7-year survival rates were 11% lower for patients with TB than for patients without TB (10% vs. 21%) (Figure 1) and 7% less for patients with DM than for patients without DM (14% vs. 21%) (Figure 2).

**Table 2:** Baseline prevalence of comorbidity among lung cancer patients and mean survival days by comorbidity and stage of lung cancer at diagnosis

**Comorbidity**	**Stage**								***P*-value**
	**I**		**II**		**III**		**IV**		
	**N = 214**		**N = 104**		**N = 399**		**N = 378**		
	%	Mean	%	Mean	%	Mean	%	Mean	
Other cancer									
No	91.6	1235	96.2	1072	94.7	718	94.4	467	-
Yes	8.4	1157	3.8	334	5.3	829	5.6	853	0.093
Tuberculosis									
No	92.5	1247	89.4	1075	90.2	743	89.4	500	-
Yes	7.5	1002	10.6	774	9.8	553	10.6	395	0.004
COPD									
No	92.1	1213	94.2	1038	95.5	726	96.3	491	-
Yes	7.9	1408	5.8	1123	4.5	685	3.7	435	<0.001
Diabetes									
No	86.9	1280	90.4	1065	88.7	752	88.1	492	-
Yes	13.1	889	9.6	836	11.3	503	11.9	466	0.008
Hypertension									
No	70.1	1209	77.9	1111	75.4	757	73.0	498	-
Yes	29.9	1275	22.1	806	24.6	622	27.0	465	<0.001

Mean: Average of survival days.

COPD: chronic obstructive pulmonary disease.

Missing data: 16 cases.

### Mortality hazard ratio

The results of the multivariate Cox proportional hazards analysis shows that men were at higher risk of death than women (HR = 1.44, 95% CI = 1.20–1.72, Table 3). The HRs were not significantly different among age groups. Patients with comorbid TB had a HR of 1.30 (95% CI = 1.03–1.65). Those with comorbid DM also had a higher risk of death (HR = 1.44, 95% CI = 1.15–1.80) during the observation period. Patients with comorbid OC, COPD or HT did not have a higher risk of death than patients without the corresponding comorbidity. The adjusted analysis showed that the stage at diagnosis had a significant association with survival. Using Stage I as the reference, the risk of death was markedly higher among patients diagnosed at advanced cancer stages (Stage II: HR =1.85, 95% CI = 1.30–2.61; Stage III: HR = 3.35, 95% CI = 2.57–4.35; Stage IV: HR = 5.63, 95% CI = 4.32–7.33).

**Table 3:** Mortality rate and multivariable Cox proportional hazards regression model measured hazard ratio (HR) and 95% confidence interval (CI) of mortality by demographic status, co-morbidity and stage of lung cancer

**Variables**	**N**	**Death**	**Person-days**	**Mortality rate**	**Rate ratio (95%CI)**		HRs* (95% CI)		***p*-value**
**Overall**	1111	738	857762	0.86					
**Gender**									
Male	822	565	615638	0.92	1.28	(1.08-1.52)	1.44	(1.20-1.72)	<0.001
Female	289	173	242124	0.71	1.00		1.00		
**Age, years**									
≤ 50	94	68	54514	1.25	1.47	(1.14-1.90)	1.29	(0.99-1.70)	0.064
51 - 60	155	98	114428	0.86	1.01	(0.81-1.26)	0.89	(0.71-1.12)	0.328
61 - 70	290	183	229726	0.80	0.94	(0.79-1.12)	1.00	(0.83-1.20)	0.994
≥ 71	572	389	459094	0.85	1.00		1.00		
**Comorbidity**									
Other Cancer									
No	1031	690	787641	0.88	1.00		1.00		
Yes	64	38	57496	0.66	0.75	(0.54-1.05)	0.77	(0.55-1.07)	0.124
Tuberculosis									
No	989	649	783186	0.83	1.00		1.00		
Yes	106	79	61951	1.28	1.54	(1.22-1.94)	1.30	(1.03-1.65)	0.030
COPD									
No	1040	696	795978	0.87	1.00		1.00		
Yes	55	32	49159	0.65	0.74	(0.52-1.06)	0.97	(0.67-1.38)	0.848
Diabetes									
No	967	633	768281	0.82	1.00		1.00		
Yes	128	95	76856	1.24	1.50	(1.21-1.86)	1.44	(1.15-1.80)	0.002
Hypertension									
No	808	534	636661	0.84	1.00		1.00		
Yes	287	194	208476	0.93	1.11	(0.94-1.31)	1.07	(0.90-1.27)	0.453
**Stage**									
I	216	73	264485	0.28	1.00		1.00		
II	106	60	110785	0.54	1.96	(1.39-2.76)	1.85	(1.30-2.61)	0.001
III	406	289	295328	0.98	3.55	(2.74-4.58)	3.35	(2.57-4.35)	<0.001
IV	383	316	187164	1.69	6.12	(4.74-7.89)	5.63	(4.32-7.33)	<0.001
*p* for trend							<0.001		

COPD: chronic obstructive pulmonary disease.

* Adjusted Hazard Ratios, obtained with statistical adjustment for all the variables listed in this table.

## Discussion

This study examined how patient characteristics and selected comorbidities are associated with survival for patients with lung cancer. Patients with comorbid TB or DM had a reduced duration of survival, and a higher mortality hazard. Younger patients had a shorter survival duration than older patients, a phenomenon has not been previously reported.

Previous studies have shown that patient demographic characteristics such as age, sex, marital status, and education are important factors associated with cancer survival [13,17,23-26]. We found sex to be significantly associated with survival in the multivariate analysis (Table 3). Men had a shorter survival duration than women and an approximately 44% increased mortality hazard. This result is consistent with other studies [16,27,28]. It is generally recognized that lung cancer survival among women is far better than that among men [27,28]. Lung cancer is biologically different in men and women. The biological characteristics and prognostic profiles of the tumor may also differ between them [26-28]. Approximately only 4.0–9.0% of women are smokers in Taiwan. Female patients are more likely to have never smoked than male patients [29,30]. Taiwanese women are also more likely than men to present with adenocarcinoma rather than squamous carcinoma of the lung [31].

Education, religion, marital status, and occupation were not found to be significantly related to survival in the adjusted analysis. Several previous studies have found a significant association between age and survival [13,23,24,26]. We found that younger patients had a shorter survival in both the crude and adjusted analyses. A further analysis using a contingency table of age by stage showed that a greater proportion of younger patients (≤ 50 years) than older patients had their lung cancer diagnosed at a late stage (47.9% vs. 30.1% in stage IV) (data not shown). This late detection is likely to explain why the younger patients had a much shorter survival duration than the oldest group of patients (580 ± 526 vs. 803 ± 693 days on average). Young patients are apparently unaware of the importance of early detection. They are also more likely to be heavy smokers [16,32].

The lung cancer survival duration was also found to be determined by the stage of disease, tumor biology and comorbidity. Comorbidity is not only an independent prognostic factor for surgical resection, but also important in host-resistance and host–tumor interaction, and has a significant role in survival [2,11-13,19].

Only a few studies have investigated the association between comorbidity and lung cancer survival. Tammemagi et al. and Battafarano et al. found support for the hypothesis that comorbidity is inversely related to survival duration [11,14]. Several studies found that TB, DM, OC, COPD and peripheral vascular disease may independently predict reduced survival duration [9,14]. Our study also found that lung cancer patients with comorbid TB or DM have a shorter survival duration across all stages of lung cancer. However, some other studies have contradictory findings. Janssen-Heijnen et al. reported that comorbidity was not a significant factor determining cancer survival [13]. Poorer survival among patients with TB or DM, even with stage at initial diagnosis held constant, may stem from less efficient immunization, anti-tumor defense systems, and multiple organ dysfunctions with these conditions. Poorer lung function, physical performance status, and nutrition, as well as lower immunization among lung cancer patients with TB may influence the available treatment choices [19,20]. Furthermore, the chronic cough caused by TB may cause patients to be ignorant their lung cancer and delay them from seeking medical treatment, thus influencing their survival.

The current clinical evaluation of treatment effectiveness and the care of lung cancer patients put more emphasis on cancer stage and tumor biology, while ignoring other patient characteristics. Comorbidities may influence the choice of treatment and treatment side effects, which are associated with patient survival. Therefore, physicians should carefully evaluate each patient’s comorbidities. In the present study, 9.7% of the lung cancer patients had TB. TB patients are at higher risk of developing lung cancer. Yu et al. found that patients with pulmonary TB are at 11-fold higher risk of developing lung cancers than those without TB [33]. Therefore, it is necessary to examine the respiratory tract symptoms of patients with TB to screen for lung cancer and improve their survival. DM is one of the world’s major chronic diseases and leading causes of death. DM patients have poor nutrition absorption, and are at risk of higher glucose and immunization, which may also lead to limited treatment choices and reduced survival [2,7-10].

## Conclusions

In conclusion, this study suggests that lung cancer patients with comorbidity, particularly DM or TB deserve more attention while undergoing cancer treatment. In addition, having a valid disease-specific instrument to measure and classify the overall severity of comorbidity is very important for improving the outcome of lung cancer care, especially for long-term survival. This study also observed shorter survival durations among young patients. More attention should be devoted to these patients, who may have had the disease diagnosed at a late stage.

Our findings reflect the importance of public health in reducing the prevalence of comorbidities and late diagnosis among lung cancer patients. However, the biological and/or appropriate therapeutic implications of comorbidities for lung cancer have not been addressed in this study. They remain important issues for future study. Further investigations focusing on caring for lung cancer patients with comorbid diseases such as TB or DM are needed to provide direction for clinicians treating patients with comorbid conditions.

## Conflicting interests

There are no potential conflicts of interest for any of the authors. All authors have no reportable conflicts.

## Authors’ contributions

C and S designed the study. C was involved in the provision of study materials and patients. S, P, T and L performed the data analysis and interpretation. C and S wrote the manuscript together with P and S. All authors read and approved the final manuscript.

## Pre-publication history

The pre-publication history for this paper can be accessed here:

http://www.biomedcentral.com/1471-2407/12/174/prepub
